# Beyond the state of the art of reverse vaccinology: predicting vaccine efficacy with the universal immune system simulator for influenza

**DOI:** 10.1186/s12859-023-05374-1

**Published:** 2023-06-05

**Authors:** Giulia Russo, Elena Crispino, Avisa Maleki, Valentina Di Salvatore, Filippo Stanco, Francesco Pappalardo

**Affiliations:** 1 Department of Health and Drug Sciences, Università degli Studi di Catania, Catania, Italy; 2 Department of Biomedical and Biotechnological Sciences, Università degli Studi di Catania, Catania, Italy; 3 Department of Mathematics and Computer Science, Università degli Studi di Catania, Catania, Italy

**Keywords:** In silico trial, Vaccine desig, Influenza, Reverse vaccinology, UISS

## Abstract

When it was first introduced in 2000, reverse vaccinology was defined as an in silico approach that begins with the pathogen's genomic sequence. It concludes with a list of potential proteins with a possible, but not necessarily, list of peptide candidates that need to be experimentally confirmed for vaccine production. During the subsequent years, reverse vaccinology has dramatically changed: now it consists of a large number of bioinformatics tools and processes, namely subtractive proteomics, computational vaccinology, immunoinformatics, and in silico related procedures. However, the state of the art of reverse vaccinology still misses the ability to predict the efficacy of the proposed vaccine formulation. Here, we describe how to fill the gap by introducing an advanced immune system simulator that tests the efficacy of a vaccine formulation against the disease for which it has been designed. As a working example, we entirely apply this advanced reverse vaccinology approach to design and predict the efficacy of a potential vaccine formulation against influenza H5N1. Climate change and melting glaciers are critical due to reactivating frozen viruses and emerging new pandemics. H5N1 is one of the potential strains present in icy lakes that can raise a pandemic. Investigating structural antigen protein is the most profitable therapeutic pipeline to generate an effective vaccine against H5N1. In particular, we designed a multi-epitope vaccine based on predicted epitopes of hemagglutinin and neuraminidase proteins that potentially trigger B-cells, CD4, and CD8 T-cell immune responses. Antigenicity and toxicity of all predicted CTL, Helper T-lymphocytes, and B-cells epitopes were evaluated, and both antigenic and non-allergenic epitopes were selected. From the perspective of advanced reverse vaccinology, the Universal Immune System Simulator, an in silico trial computational framework, was applied to estimate vaccine efficacy using a cohort of 100 digital patients.

## Background

Reverse vaccinology (RV) conjugated with computational modeling and simulation can accelerate and facilitate the vaccine discovery pipeline. RV approaches have been developed to take advantage of pathogens' genome sequence to narrow the number of antigens for selecting the most suitable candidate. To this aim, portions of each protein candidate should be predicted using bioinformatic software or retrieving information from biological databases and web servers. RV tools score predicted characteristics based on a manually chosen filter and rank. For example, to control a new possible pandemic due to genome recycling, RV can assist in accelerating the discovery of potential new targets that can be used in a vaccine formulation (or therapeutic) [1]. However, a multi-epitope vaccine computer-aided design is the most frequent immunoinformatic approach in the RV pipeline. Multi-epitope vaccine workflow is mainly categorized into four stages comprising *(i)* data collection and preparation; *(ii)* selection of those proteins that are naturally exposed to the immune system; *(iii)* epitope prediction; and *(iv)* computational analysis and verification of vaccine candidate [2].

Although the RV now includes many bioinformatics tools and processes compared to when it was first introduced, it is still unable to predict the efficacy of a suggested vaccine formulation. Hence it also lacks the possibility to predict therapeutic failures.

Nowadays, climate change, particularly the melting of glaciers, represents critical issues. Ice is a reservoir of microorganisms, including fungi, bacteria, and viruses, which can be preserved in glaciers for over 100,000 years [3–8]. Environmental ice provides a mechanism for the surviving and recycling of microorganisms, which Rogers et al. called "*genome recycling*" [9]. It consists of the microorganisms traveling into the atmosphere and then deposition onto the glacier, where they are entrapped for days, years, centuries, millennia, or longer.

Influenza A virus survives freezing [10], is known to be present in frozen lakes frequented by migratory aquatic birds, and has a high spill-over capability. Influenza viruses belong to the *Orthomyxoviridae* family, and they are classified into A, B, and C subtypes, depending on the differences in their nucleoprotein (NP) and in their matrix protein (M1) [11]. Influenza A virus is an RNA virus that rapidly evolves thanks to the mechanisms of antigenic drift and antigenic shift [12]. Hemagglutinin (HA) and neuraminidase (NA) are the two most important structural and surface proteins, and to date, 18 HA and 11 NA are known [13]. The HA protein attaches to sialic-acid-terminating surface receptors and actively induces the endosome membrane to fuse during virus entry.

In contrast, the NA protein demonstrates the enzymatic activity of the influenza virus to remove sialic acid and reduce extracellular virion aggregation and superinfection. Antibodies to the NA protein are protective individually in animal challenge studies. However, the neuraminidase antibodies protect differently than the hemagglutinin protein antibodies [14, 15]. HA and NA proteins can both provoke cellular and humoral immune systems.

Influenza A virus has caused multiple pandemics over the past century, and some of them were caused by reassorted viruses, which had a combination of avian and human genes [15]. H5N1 strain has evolved throughout history, giving rise to different variants and causing the onset of zoonotic epidemics and human infections called "avian influenza." There is little evidence of human-to-human transmission, suggesting that this virus is not fully adapted to the human host [15]. Still, there is evidence of mutations accumulated by H5N1 that have made it more virulent and deadly in mammals [16]. The possibility of H5N1 being recycled from environmental ice, along with further mutations that could occur during human replication or a possible reassortment with a human virus, could produce a more virulent and contagious virus for humans, opening the door to potential pandemics.

To fill the gap that affects RV, here we present an advanced immune system simulator that enriches the RV pipeline with the possibility of testing the efficacy of a vaccine formulation. In particular, as a working example, we propose a potential vaccine formulation against influenza H5N1. It is fully designed by combining different sets of programs presently used in RV (e.g., databases, software, and tools) with an immune system simulator that is in charge of predicting the efficacy of the proposed vaccine, also optimizing the dosage.

## Material and methods

The Universal Immune System Simulator (UISS) is an agent-based model that accurately simulates the hallmarks of the human immune system [16].

UISS uses a multi-layer approach that considers three layers:

- The physiological response of the immune system to a *non-self* (or *self* in the presence of immune system impairment) entity (physiology layer);
- The dynamics related to the progression of the disease (disease layer);
- Eventually, the effects induced by different treatments on the control of the disease (treatment layer).

The multi-layer approach allows UISS to be easily adapted and extensively applied to a large set of biological scenarios, as an in silico trial [17], to predict the disease progression and the effect of immunotherapies [18–23].

UISS–FLU is a specific disease layer implementation added to the UISS general framework that aims at simulating the dynamics of the immune system response to the H5N1 influenza A infectious disease. Moreover, UISS-FLU has also been added with the treatment layer able to simulate and predict the efficacy of a potential vaccine formulation.

**Fig. 1:**
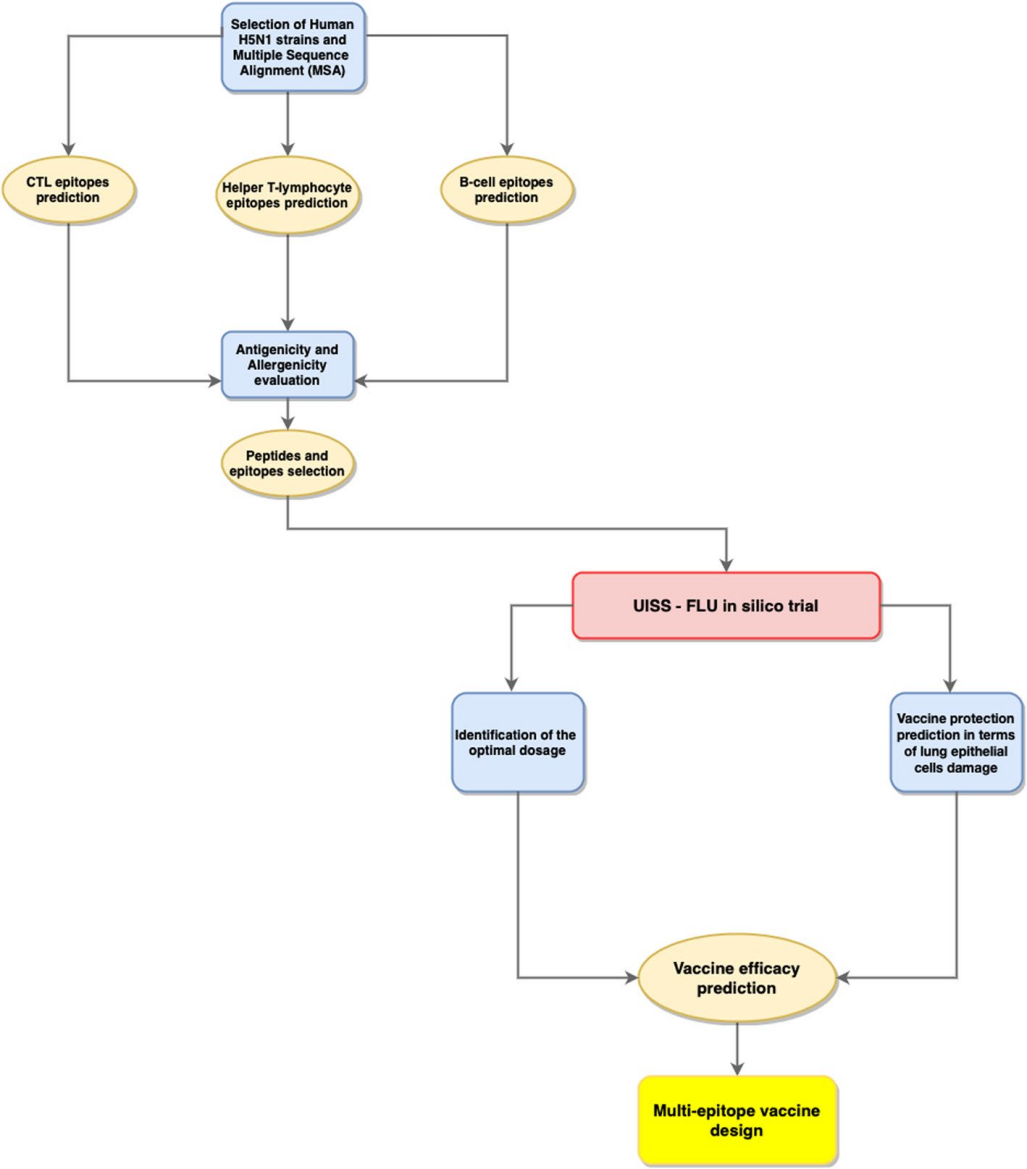
Workflow of the proposed advanced RV pipeline for multi-epitope vaccine design. After retrieving the H5N1 strain sequence from the NCBI database and performing Multiple Sequence Alignment (MSA), we predict CTL, Helper T-lymphocyte, and B cell epitopes using specific bioinformatic tools to evaluate their antigenicity and allergenicity levels and select the best epitopes. Finally, UISS-FLU is run to predict the efficacy of the multi-epitope vaccine formulation

To go beyond the state of the art of RV, we propose this advanced workflow that can also predict the efficacy of the proposed vaccine formulation in a disease-compliant environment. The workflow, depicted in Fig. 1, consists of:

- Selection of FASTA sequence of HA and NA proteins from different human H5N1 strains;
- Multiple Sequence Alignment (MSA);
- Prediction of Cytotoxic T-lymphocyte epitopes (CTL);
- Prediction of Helper T-lymphocyte epitopes;
- Identification of linear B-cell epitopes;
- Evaluation of antigenicity and allergenicity of all predicted epitopes;
- Efficacy prediction of selected peptides through UISS-FLU modeling and simulation platform.

This workflow may be further enhanced using the multiple methods integration. For example, the idea suggested in [24] can be adopted. However, the main aim of this work is to present a way to predict the efficacy of an in silico*-designed* vaccine candidate in a disease-centered simulation environment.

### Selecting sequences of the most common H5N1 strains in humans and multiple sequence alignment (MSA)

The HA protein sequences of four H5N1 strains (A/Thailand/676/2005, A/Hong Kong/481/97, A/Hong Kong/213/2003, A/Viet Nam/1203/2004) and the NA protein sequences of other four H5N1 strains [A/Thailand/676/2005(H5N1), A/Hong Kong/213/2003(H5N1), A/Vietnam/CL115/2005(H5N1), A/Vietnam/CL36/2004(H5N1)] that were widespread around the world from 1997 to 2005, were retrieved from the National Center for Biotechnology Information (NCBI) database. Then, we performed the multiple sequence alignment (MSA) for both HA and NA sequences to obtain a consensus sequence that usually includes conserved residues, amino acids, or nucleotides. MSA provides a computational environment to analyze the homology and evolutionary correlation between the sequences investigated. We used ClustalOmega [25], while the results were analyzed through JalView [26]. ClustalOmega is a program that allows doing multiple sequence alignments, generating alignments between three or more sequences. We uploaded the obtained sequences on JalView software, a free MSA editing, visualization, and analysis program. It is a web-based application that may also be installed locally and allows in-depth sequence analysis required when one learns about novel protein or RNA sequence groups, and how their sequence is related to their structure and function.

### Prediction of CTL epitopes

Cytotoxic T-lymphocyte (CTL) epitopes were predicted using the web-based tool called NetCTL 1.2 Server.^1^ NetCTL 1.2 predicts human CTL epitopes in any protein by combining proteasomal C terminal cleavage predictions, TAP transport efficiency, and MHC class I binding affinity predictions. We inserted the consensus sequences in FASTA format for the CTL epitopes prediction. We selected the human leukocyte antigen (HLA) Class I supertypes primarily involved in recognizing influenza epitopes: A1, A3, A24, and B7 alleles [27]. The weight on C terminal cleavage, TAP transport efficiency, and the threshold for epitope identification were set to default values of 0.15, 0.05, and 0.75, respectively.

### Prediction of Helper T-lymphocytes epitopes

The Helper T-lymphocytes epitopes were predicted using the NetMHCIIpan 4.0 Server,^2^ which can predict the affinity binding score between a peptide and any MHC class II molecule [28]. Then, we uploaded the consensus sequences and selected the species "HighQ-DRB" with the alleles DRB1_0101, DRB1_0401, and DRB1_0402, respectively, as well as the species "DP" with the allele HLA-DPA10401-DPB10401.

### Identification of linear B-cell epitopes

The linear B-cell epitopes were predicted using the BepiPred Linear Epitope Prediction.^3^ It is a web server able to predict B-cell epitopes from antigen sequences. It is based on a random forest algorithm trained on epitopes from antibody-antigen protein structures. A random forest is a supervised machine learning algorithm based on the predictions of the decision trees algorithm [29]. The random forest algorithm generates a "forest" of decision trees trained to predict the outcome of a specific event by taking the average or mean of the output from the various trees in the forest. The higher the number of trees, the higher the prediction's precision. BepiPred takes protein sequences in FASTA format as input. It produces a table showing each residue with related scores and a significance threshold indicating if they are predicted to be part of an epitope as output. The global antigenicity value for the entire peptide (B), representing its ability to be specifically recognized by the antibodies generated due to immune response, is calculated upon the scores of single epitopes as follows:

$$B =\frac{{\sum }_{ i=1}^{ i=l}{ }_{{a}_{i}} }{l}$$

where ${a}_{i}$ is the antigenicity value for each residue in the peptide, and $l$ is the total number of residues of the peptide. Peptides showing $B\ge 0.4$ were considered antigenic and used in the next steps of the analysis.

### Evaluation of antigenicity and allergenicity of collected CTL, Helper T-cells, and B-cell epitopes

Antigenicity is the capacity of an antigen to combine with the end products of the immune response, i.e., the ability of an antigen to induce an immunological response when the human body encounters it [30]. Allergenicity^4^ refers to the capacity of an antigen to induce an anomalous immune response, which usually does not result in a protective effect but may cause function disorder or tissue damage. The antigenicity of all high-binding predicted CTL, Helper T-cells, and B-cells epitopes was evaluated using VaxiJen v2.0.^5^ VaxiJen is a server for predicting protective antigens, which is alignment-independent. It was designed to perform antigen classification based only on the physicochemical properties of proteins without using sequence alignment. VaxiJen uses auto cross-covariance (ACC) to transform protein sequences into fixed-length vectors. We submitted one peptide at a time among the selected high-binding epitopes to evaluate their antigenicity. The target organism was set to a virus, and the threshold was the default one, set to 0.4 for our model. Then, to evaluate the allergenicity of selected high-binding epitopes, we used AllerTOP v.2.0.^6^ AllerTop v.2.0 is based on translating protein sequences into uniform equal-length vectors using ACC [31] in the same way as VaxyJen. We inserted the sequence of each strong binder peptide found in the previous three steps and evaluated their allergenic potentiality. Finally, we collected all the epitopes found to be both antigenic and non-allergenic [30].

### *UISS-FLU *in silico* trial for the selected epitopes*

UISS-FLU is a specific implementation of the influenza disease layer in the UISS modelling and simulation platform. It can simulate the dynamics of the infection along with the disease progression, analyzing the lung compartment and, in particular, the lung epithelial cells as an H5N1 preferential target. In particular, UISS-FLU considers the immunological machinery for computing the affinity receptor binding of B and T cells. To this aim, we implemented a mathematical infrastructure through the function *Affinity.* It takes as an input the parameter *m* representing the affinity of an m-bit match of each component of the vector of length bits. Since we calculated the affinity scores for B cells and T cells epitopes, we imported such scores directly into the affinity vector for the epitopes and peptides, matching the residues we would like to test. We created cohorts of 100 digital patients to test the selected formulations as described in [32]. Basically, all real patients are characterised by a series of biological and pathophysiological parameters which uniquely identify them by associating each of them with a specific immunological profile. To generate the cohort of virtual patients to be submitted to the simulator, and still keep the biological diversity unchanged, it is necessary to assign a single value for each parameter included in the vector of features. In this way, varying from time to time one of the above values, you get many different vectors of features representing different digital patients, all equally plausible and acceptable from the biological and pathophysiological point of view. Below, the mechanism of creating vector of features is formally explained, as well as the technique used to take into account all possible biological correlations between them. In particular, a vector of features contains 22 input variables, for which a number of correlations equal to 22 × 21/2 = 231 is also calculated.

This approach is based on current mathematical biology consensus and uses a Gaussian distribution to represent the population. The vector $f=\left\{{f}_{1}, {f}_{2},\dots {f}_{d}\right\}$ follows a d-variate Gaussian distribution with the following probability density function. This approach is based on current mathematical biology consensus and uses a Gaussian distribution to represent the population. Formally, the vector $f=\left\{{f}_{1}, {f}_{2},\dots {f}_{d}\right\}$ follows a d-variate Gaussian distribution with the following probability density function:

$${N}_{d}\left(f\left|\mu ,\Sigma \right|\right)=\frac{{\left|\Sigma \right|}^{-\frac{1}{2}}}{{\left(2\pi \right)}^\frac{d}{2}}\mathrm{exp}\left[-\frac{1}{2}{\left(f-\mu \right)}^{^{\prime}} {\Sigma }^{-1}\left(f-\mu \right)\right]$$

where $\mu$ is the mean, and $\Sigma$ is the covariance matrix, defined as follows:

$$\Sigma = \left( {\begin{array}{*{20}c} {\sigma_{1}^{2} } & {\sigma_{12} } & \cdots & {\sigma_{1d} } \\ {\sigma_{21} } & {\sigma_{2}^{2} } & \cdots & {\sigma_{2d} } \\ \vdots & \vdots & \ddots & \vdots \\ {\sigma_{d1} } & {\sigma_{d2} } & \cdots & {\sigma_{d}^{2} } \\ \end{array} } \right),$$

where ${\sigma }_{ij}=Cov\left({x}_{i}, {x}_{j}\right)$ and it is related to the correlations through the following formula:

$$Cor\left({x}_{i}, {x}_{j}\right)={\rho }_{ij}=\frac{{\sigma }_{ij}}{\sqrt{{\sigma }_{i}^{2}{\sigma }_{j}^{2}}}$$

We can calculate the covariance between two inputs by measuring their correlation. The elements on the diagonal of the covariance matrix, ${\sigma }_{i}^{2}$, represent the marginal variances of each element, ${f}_{i}$, and ${\mu }_{i}$ represents the corresponding marginal mean.

In general**,** we assume that ${f}_{s}$ is the vector of pre-defined features related to each patient, so that $f=\left\{{f}_{s}, {f}_{r}\right\}$, where ${f}_{s} \in {\mathbb{R}}^{d-q}$ and ${f}_{r} \in {\mathbb{R}}^{q}$ is the vector of free features.

A treatment layer was also implemented into UISS-FLU to simulate the vaccine formulation. In particular, for the proposed multi-epitope vaccine, we modeled the liposomal (LP) structure recognized as one of the most common and effective vaccine delivery systems.

We designed the in silico trial with a 100 patients virtual cohort to obtain sufficient statistical power regarding immunological variability. UISS-FLU considers and implements three levels of stochasticity, which, in turn, are related to the biological diversity simulation. The first one deals with immunological repertoire. UISS-FLU can simulate different MHC class I and II that influence the generation of the T and B repertoire shape. The second one implements the probability associated with different allelic expression within HLA molecules that is strictly correlated to the effective presentation of antigens by HLA class I molecules to CD8^+^ T cells, required for viral elimination and generation of long-term immunological memory. The last one is related to the stochasticity involved in the initial conditions of the simulation itself. The stochastic nature of initial conditions can allow for varying the initial disposition of entities in the compartment to exclude dependencies that may affect the simulation (i.e., provide a statistical error quantification on initial conditions). The 100 virtual patients have been set to reproduce the HLA "HighQ-DRB" with the alleles DRB1_0101, DRB1_0401, and DRB1_0402, as well as the species "DP" with the allele HLA-DPA10401-DPB10401, for maximizing vaccine coverage. Among the 100 virtual patients, we varied the initial conditions and the probability associated with allelic expression within HLA to guarantee the avoidance of any statistical issues related to probabilistic perturbance.

We simulated different scenarios, i.e.:

1. Influenza virus challenge only virtual cohort.
2. Multi-epitope vaccine administered without virus challenge virtual cohort.
3. One injection of multi-epitope vaccine (with a different dosage ranging from 5000 to 1,500,000 LP per ml) and influenza challenge at 40, 60, and 120 days.
4. One injection of multi-epitope vaccine (with a dosage of 500,000 LP per ml), a booster dose at 90 days (with the same dosage), and then an influenza challenge at 120 days.

To analyze the simulation results of the 100 patients' virtual cohorts, we used MetricUISS [33]. MetricUISS is a tool developed in Python3, specifically designed for the statistical analysis of large amounts of data generated by UISS. Assessing disease severity in influenza and consequently evaluating the vaccine efficacy based on the number of infected cells alone may not provide a comprehensive measure. Various factors, including viral load, host immune response, and clinical symptoms, influence disease severity in influenza. Even if the simulation infrastructure cannot evaluate clinical symptoms directly, it can quantitively assess the viral load, the number of infected respiratory epithelial cells, and the immune system response. In Results section, also looking at the good clinical and laboratory practices to evaluate vaccine efficacy, we highlighted as the overall quantification of the involved biomarkers measuring disease severity and immune system response is revealing a good protection from disease.

## Results

### Selecting sequences of the most widespread H5N1 strains in humans and multiple sequence alignment (MSA)

Multiple sequence alignment (MSA) was performed through ClustalOmega on both HA and NA protein sequences for each H5N1 strain retrieved on the NCBI database. After completing MSA, the consensual sequences consisting of 575 residues for HA and 468 for NA, were obtained by JalView.

### Prediction of CTL epitopes

NetCTL 1.2 Server was used to predict 9-mer long CTL epitopes. Two different thresholds indicate the weak and the strong binding peptides; the threshold with the value of 0.500 represents a potent binding peptide, whereas the threshold with the value of 2.000 shows a weak binding peptide. We selected the human HLA Class I supertypes A1, A3, A24, and B7 most involved in recognizing influenza epitopes. In addition, 19 high-binder CTL epitopes of HA and 12 of NA protein were collected for further analysis.

### Prediction of Helper T- lymphocytes epitopes

NetMHCIIpan 4.0 Server was used to predict 15-mer long Helper T-lymphocytes epitopes. Specific thresholds for strong and weak binders are shown in terms of % Rank, which is the percentile of predicted binding affinity compared to the distribution of affinities calculated on a collection of random natural peptides. If the peptide is among the selected threshold for strong binders (which we set to 1%), it will be classified as a strong binder. If the % Rank is higher than the threshold for strong binders but lower than the set threshold for weak binders (which we set to 5%), the peptide will be classified as a weak binder. We collected 23 high-binder epitopes of HA and 29 of NA protein for more analysis.

### Identification of linear B-cell epitopes

BepiPred Linear Epitope Prediction 2 was used to gain linear B-cell epitopes. Only the epitopes with a score above the threshold, whose value is 0.4, were selected. As a result, we retrieved 11 epitopes of HA and nine epitopes of NA protein with a length between 10 and 40 amino acids.

### Evaluation of antigenicity and allergenicity of selected CTL, Helper T-lymphocytes, and B-cell epitopes

The antigenicity and allergenicity of the selected epitopes were evaluated using VaxiJen v2.0 and AllerTOP v.2.0 servers. We collected all the peptides found to be both antigenic and non-allergenic, including two CTL epitopes, nine Helper T-lymphocytes epitopes, and three B-cell epitopes for HA protein, while five CTL epitopes, six Helper T-lymphocytes epitopes, and one B-cell epitope for the NA protein.

### *UISS-FLU *in silico* trial of the selected epitopes*

Applying the first steps described in the wortkflow depicted in Fig. 1, we selected the best epitopes accordingly to the scores released by the tools described above. Table 1 shows the best ranked epitopes.

**Table 1:** Selected CD8, CD4 and B cell epitopes accordingly to the score computed by NetCTL, NetMHCIIpan and BepiPred predictors

ID	Allele	Epitope	Antigenicity	Allergenicity	Score
*MHC-I (CD8 T cells)*					
CTL1	HLA-A*01:01	KSDQICIGY	Antigenic	Non-allergen	0,889,895
CTL2	HLA-A*24:02	LYDKVRLQL	Antigenic	Non-allergen	0,736,697
CTL3	HLA-B*07:02	CPYQGKSSF	Antigenic	Allergen	0,917,161
*MHC-II (CD4 T cells)*					
HTL1	DRB1_0101	EWSYIVEKANPANDL	Antigenic	Non-allergen	0.994155
HTL2	DRB1_0101	SYIVEKANPANDLCY	Antigenic	Non-allergen	0.877016
HTL3	DRB1_0101	PTTYISVGTSTLNQR	Antigenic	Non-allergen	0.793413
HTL4	DRB1_0101	EFFWTILKPNDAINF	Antigenic	Non-allergen	0.862769
HTL5	DRB1_0402	KIQIIPKSSWSSHEA	Antigenic	Non-allergen	0.834586
HTL6	DRB1_0402	IQIIPKSSWSSHEAS	Antigenic	Non-allergen	0.642752
HTL7	HLA-DPA10401-DPB10401	GRMEFFWTILKPNDA	Antigenic	Non-allergen	0.26179
HTL8	HLA-DPA10401-DPB10401	RMEFFWTILKPNDAI	Antigenic	Non-allergen	0.25399
HTL9	DRB1_0101	PEWSYIVEKANPAND	Antigenic	Non-allergen	0.995068
HTL10	HLA-DPA10401-DPB10401	GRMEFFWTILKPNDA	Antigenic	Non-allergen	0.261798
HTL11		DAINFESNGNFIAPE	Antigenic	Non-allergen	0.58238

ID	Epitope	Antigenicity	Allergenicity	Score
*B_HA*				
B_HA1	QRLVPRIATRSKVNGQSG	Antigenic	Non-allergen	0.569
B_HA2	GAINSSMPFHNI	Antigenic	Non-allergen	0.582
B_HA3	MESVRNGTYDYPQYSEEARLKREEI	Antigenic	Non-allergen	0.5354
*B_NA*				
B_NA1	DTVGWSWPDGAELPF	Antigenic	Non-allergen	0.53013333
B_NA2	LLNDKHSNGTVKDRSP	ANTIGEN	Allergen	0.637

Antigenicity and allergenicity was also evaluated through VaxiJen and AllerTOP servers

UISS-FLU was then run over single patient simulations on different formulations, using both HA and NA proteins for CTL, Helper-T-cells, and B epitopes. At the end of this single-patient simulation cycle, to select the most promising formulation, we performed new UISS-FLU simulations on a cohort of 100 virtual patients. MetricUISS was finally employed to recover overall results, finding that the best epitopes formulation was the following:

$$LYDKVRLQL\;\left( {MHC - I} \right) + DAINFESNGNFIAPE\left( {MHC - II} \right) + LLNDKHSNGTVKDRSP\;\;\left( B \right)$$

with a 500,000 LP per ml dosage and a booster at 90 days. These epitopes show an affinity score of 0.737 for MHC-I, 0.582 for MHC-II, and 0.637 for B, respectively.

For all the simulated scenarios, we obtained a comprehensive overview of the following different immune system dynamics:

- Lung epithelial infected cells and total lung epithelial cells population levels.
- IgM, IgG, and IgA concentration levels.
- IL-1, IL-2, IL-6, IL-12, IFN-γ, and TNF-α concentration levels.
- Neutrophils, MHC-II antigen-presenting macrophages, MHC-I antigen-presenting dendritic cells, and MHC-II antigen-presenting dendritic cells population levels.
- Activated CD4 + Th1 cells, activated CD8 + T cells, total memory B cells, and total memory Th1 cells population levels.

The complete collection results for all the conditions can be found in Additional file (data availability section). However, for this study, only the results of the following scenarios have been included in the manuscript:

- One injection of multi-epitope vaccine (with a dosage of 500,000 LP per ml) at time 0, and a booster dose after 90 days (with same dosage), with no influenza challenge exposure, aimed at evaluating the effect of the multi-epitope vaccine on the immune system.
- One injection of multi-epitope vaccine (with a dosage of 500,000 LP per ml) at time 0, a booster dose at 90 days (with the same dosage), and then influenza challenge exposure at 120 days to evaluate the immune system response to H5N1 influenza virus after two doses administration of the multi-epitope vaccine.
- Immune system response to influenza virus after two doses compared to the immune response after only one dose of multi-epitope vaccine.

#### Immune system response after two doses of multi-epitope vaccine

Using UISS-FLU, we simulated a cohort of 100 virtual patients. Each virtual patient receives two doses of our multi-epitope vaccine, one at day 0 and the second 90 days after, without any previous challenge exposure to the H5N1 influenza virus.

**Fig. 2:**
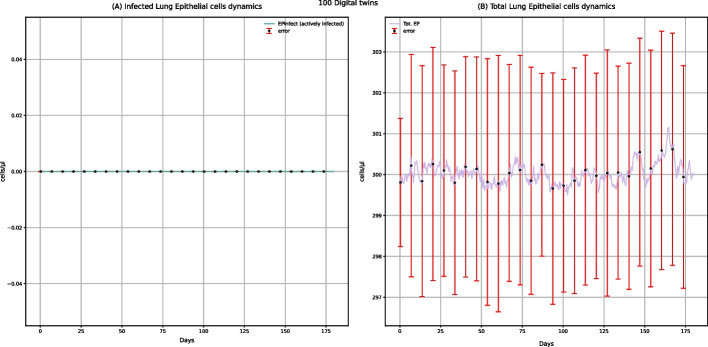
UISS-FLU in silico prediction of infected lung epithelial cells (panel **A**) and total lung epithelial (panel **B**) cells population levels. In addition, population-averaged lung epithelial cells dynamics after the first dose of the multi-epitope vaccine administered at time 0 and the second one after 90 days are depicted. Red error bars represent the standard deviation

Among all the plots produced by UISS-FLU, we obtained the ones representing the dynamics of infected and total epithelial cells (Fig. 2). These plots show that the vaccine does not affect the lung epithelial cells because the level of infected lung epithelial cells remains 0, and the number of total lung epithelial cells remains relatively constant over time. However, the dynamics of the number of lung epithelial cells after a challenge with the H5N1 influenza virus, both in digital patients with (Fig. 7) and without vaccine administration (Fig. 12), is different: generally, after H5N1 infection, an increase in the number of lung infected epithelial cells, as well as a strong reduction of the number of total lung epithelial cells, is observed. Moreover, in unvaccinated digital patients, the H5N1 influenza challenge induces a decrease of about 60% in total epithelial lung cells (Fig. 12B), in agreement with the data presented in other experimental studies concerning the influenza infection kinetics in unvaccinated humans [34]. These results demonstrate that UISS-FLU correctly simulates the effect of both vaccine administration and the H5N1 influenza virus on lung epithelial cells.

**Fig. 3:**
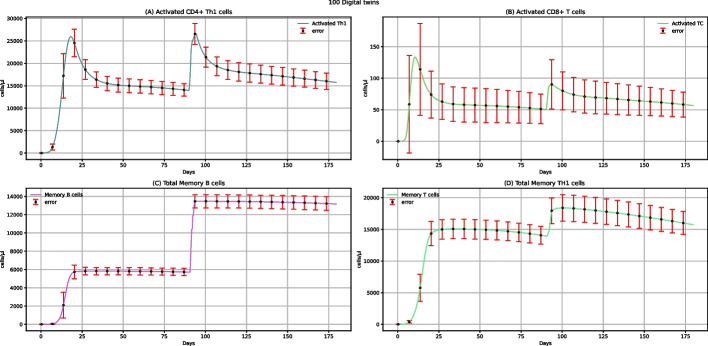
UISS-FLU in silico prediction of activated CD4 + Th1, activated CD8 + T, total memory B, and total memory Th1 cells population levels. Population averaged immune system dynamics after the first dose of the multi-epitope vaccine administered at time 0 and the second one after 90 days are depicted. Red error bars represent the standard deviation

Figure 3 shows the dynamics of activated CD4 + Th1 cells, activated CD8 + T cells, total memory B cells, and total memory Th1 cells. Vaccine administration does not affect the CD8 + T cells but can increase the activated CD4 + T cells, especially when administering two doses. Each dose increases total B memory cells (Fig. 3C) and Th1 memory cells (Fig. 3D). After the administration of two doses of vaccine, the level of these cells increases more, indeed.

**Fig. 4:**
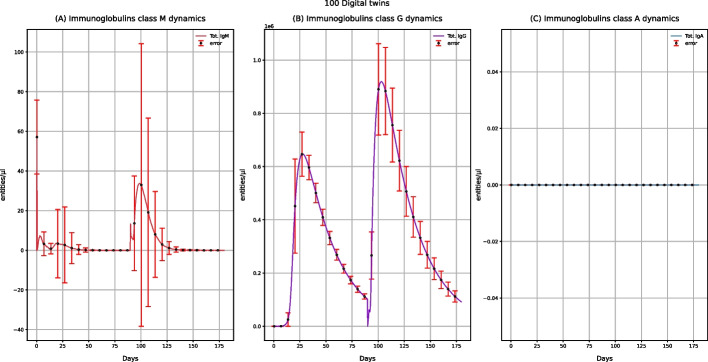
UISS-FLU in silico prediction of IgM, IgG, and IgA concentration levels. Population averaged immune system dynamics after the first dose of the multi-epitope vaccine administered at time 0 and the second one after 90 days. Red error bars represent the standard deviation

Figure 4 describes the concentration levels of IgM, IgG, and IgA. For IgG (Fig. 4B), after administering the first dose, we observe an increasing peak, while after the booster administration, the IgG peak will enormously increase. Vaccine administration also affects the IgM concentration levels, as in the plots, we have one peak after the first dose administration and another higher after booster administration (Fig. 4A). IgA's value remains equal to 0 (Fig. 4C) as they are tissue immunoglobulins, and the vaccine does not affect tissues.

**Fig. 5:**
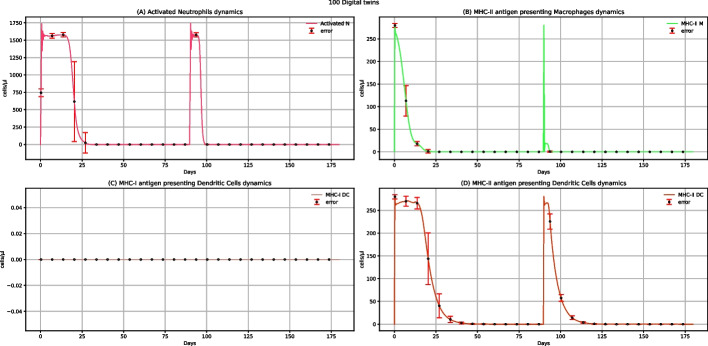
UISS-FLU in silico prediction of activated neutrophils, MHC-II antigen-presenting macrophages, MHC-I antigen-presenting dendritic cells, and MHC-II antigen-presenting dendritic cells population levels. Population averaged immune system dynamics after the first dose of multi-epitope vaccine administered at time 0 and the second one after 40 days are depicted. Red error bars represent the standard deviation

Figure 5 represents neutrophils, MHC-II antigen-presenting macrophages, MHC-I antigen-presenting dendritic cells, and MHC-II antigen-presenting dendritic cells dynamics after administering two doses of multi-epitope vaccine. Except for MHC-I antigen-presenting dendritic cells, whose value is 0 (Fig. 5C), for the other three entities (Fig. 5A, B, and D), we observe two peaks related to the increase in the number of cells after the administration of each dose, showing that the vaccine administration can elicit an immune response. MHC-I antigen-presenting dendritic cells value is 0 because when dendritic cells present the antigen in class I, they nibble infected cells that are not present if a challenge with the virus is missing.

**Fig. 6:**
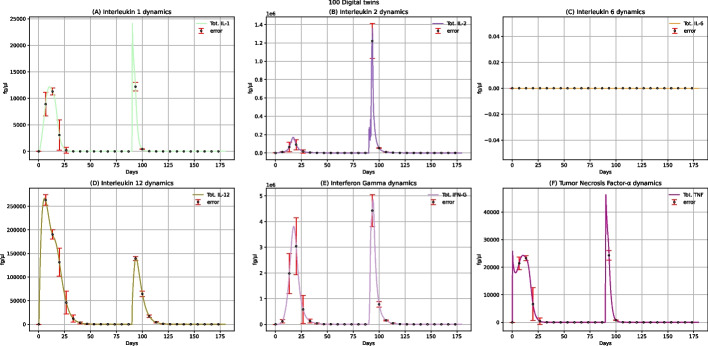
UISS-FLU in silico prediction of IL-1, IL-2, IL-6, IL-12, IFN-γ, and TNF-α concentration levels. Population-averaged immune system dynamics are depicted after the first dose of the multi-epitope vaccine administered at time 0 and the second one after 40 days. Red error bars represent the standard deviation

The dynamics of the main cytokines involved in the immune response to the multi-epitope vaccine are shown in Fig. 6. A peak characterizes IL-1 and TNF-α concentration levels after administering the first dose and a second higher peak after the booster administration (Fig. 6A and F). As IL-1 and TNF-α are known to induce an increase in body temperature [35], we can affirm that the proposed multi-epitope vaccine could cause fever, especially after administering the second dose. Generally speaking, observing IL-1, IL-2, IFN-γ, and TNF-α concentrations levels, we can also notice two peaks after the two doses of administration (Fig. 6A, B, E, F); this means that the vaccine can elicit the immune response also by producing these anti-inflammatory cytokines.

#### Immune system response to H5N1 influenza virus after two doses of multi-epitope vaccine

We also simulated the infection of a cohort of 100 in silico patients vaccinated with two doses of the multi-epitope vaccine proposed (respectively at time 0 and 90 days), obtaining a specific immune system response that allows us to evaluate the ability of the vaccine to protect individual patients from H5N1 influenza infection.

**Fig. 7:**
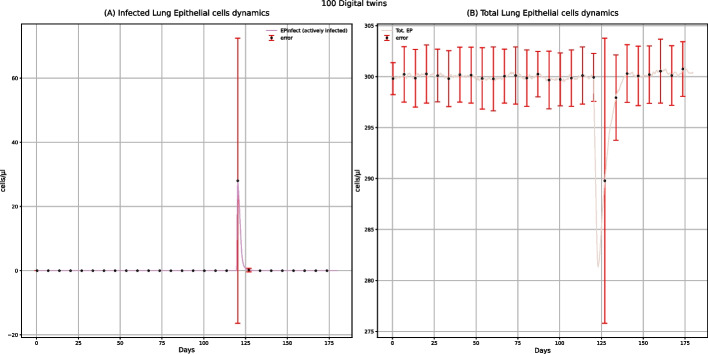
UISS-FLU in silico prediction of infected lung epithelial cells (panel A) and total lung epithelial cells (panel B) population levels. Population averaged lung epithelial cells dynamics after the first dose of the multi-epitope vaccine administered at time 0, the second at time 40, and the challenge exposure to the H5N1 virus at 120 days are depicted. Red error bars represent the standard deviation

The results coming from the dynamics of lung epithelial cells after the multi-epitope vaccine administration and challenge with the H5N1 influenza virus (Fig. 7) are different from those describing the same dynamics after only one administration of the multi-epitope vaccine (Fig. 2). The observed peaks in Fig. 7 show that the infection on these cells is caused by the infection itself and not by administering the vaccine. In Panel A, the peak with an upward trend represents the number of infected lung epithelial cells, which increases after the infection. As a demonstration of the protective ability of the multi-epitope vaccine, this peak is shorter than the one reported in the lung-infected epithelial cells in unvaccinated patients after influenza infection (Fig. 12). In Panel B, the peak with a downward trend represents the total lung epithelial cells and decreases when the virus infects those cells. Moreover, comparing this plot to the one representing the lung infected epithelial cells in unvaccinated patients after influenza infection (Fig. 12), the higher number of total lung epithelial cells after administration of two doses of vaccine is another demonstration of the protective effect of the multi-epitope vaccine itself.

**Fig. 8:**
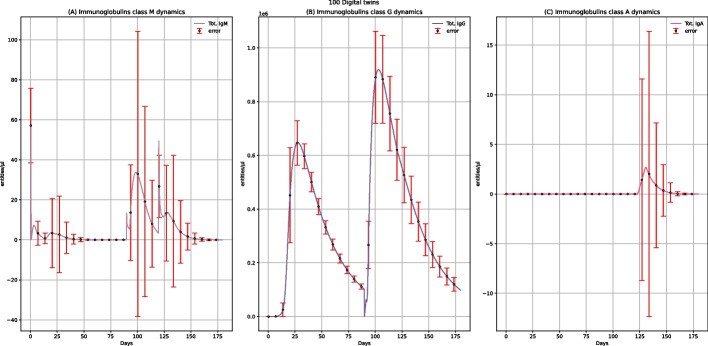
UISS-FLU in silico prediction of IgM, IgG, and IgA concentration levels. Population averaged immune system dynamics after the first dose of multi-epitope vaccine administration at time 0, the second at time 40, and the challenge exposure to the H5N1 virus at 120 days are depicted. Red error bars represent the standard deviation

The differences between the results describing the immunoglobulins dynamics in vaccinated people after the influenza challenge at 120 days (Fig. 8), rather than without the influenza challenge (Fig. 4), allow us to understand better the vaccine's effect on the humoral immune response. The IgG concentration level is almost the same in both conditions with (Fig. 8B) and without (Fig. 4B) challenge with the H5N1 virus; this indicates that IgG dynamics levels increase primarily thanks to the vaccine administration. Plots reporting IgM levels show two identical rising peaks at 40 and 90 days in both conditions (Fig. 4A, and Fig. 8A, respectively) after the administration of each dose of vaccine; however, in vaccinated patients after challenge exposure with influenza virus (Fig. 8A), another peak at 120 days is present, showing that the challenge with virus induces further production of IgM. While the IgA levels are equal to 0 after only two doses of vaccine (Fig. 4C), the challenge with the H5N1 virus leads to an increase in their level (Fig. 8C); this should be explained by the fact that IgA is tissue immunoglobulins, and while the vaccine does not affect tissues, the virus does. Furthermore, in the plot describing the IgA levels in digital patients challenged with the influenza virus without previous vaccine administration (see Additional file: Fig. 22, data availability section), there is also an increase in IgA at the time of challenge. Still, the concentration is higher than in vaccinated subjects. Precisely, this lower height of the IgA levels in vaccinated people shows how the vaccine can be able to reduce IgA production.

**Fig. 9:**
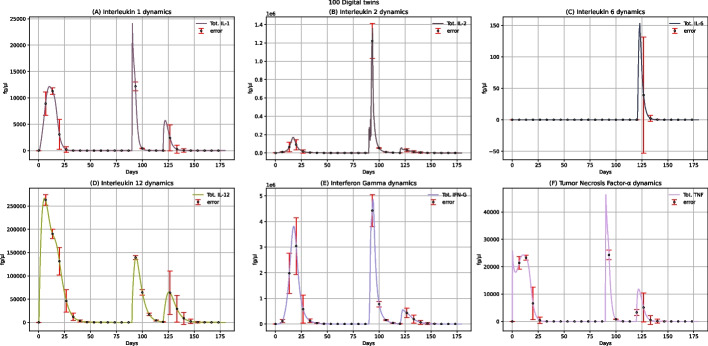
UISS-FLU in silico prediction of IL-1, IL-2, IL-6, IL-12, INF-γ and TNF-α concentration levels. Population averaged immune system dynamics after the first dose of the multi-epitope vaccine administered at time 0, the second one after 40, and the challenge exposure to the H5N1 virus at 120 days are depicted. Red error bars represent the standard deviation

Plots in Fig. 9 show the IL-1, IL-2, IL-6, IL-12, INF-γ, and TNF-α concentration levels after administering two doses of multi-epitope vaccine at 40 and 90 days, respectively, along with the challenge exposure with H5N1 virus at 120 days. Focusing on IL-1 and TNF-α, we have already reported that their level increases after vaccine administration (Fig. 6A and F). Hence we observed fever as the main adverse reaction to the vaccine. Furthermore, after the challenge with the H5N1 virus at 120 days, another peak both for IL-1 and TNF-α (Fig. 9A and F) can be observed: this latter presents a lower height compared to the ones related to the increase of the two cytokines linked to the administration of the vaccine, meaning that this multi-epitope vaccine protects patients against fever after the infection.

The high peak at 120 days present in IL-6 dynamics (Fig. 9C) shows that the infection induces IL-6 production, conversely to the vaccine (Fig. 6C).

After the challenge with the H5N1 virus, comparing vaccinated patients to the unvaccinated ones, the results show a markable reduction of ING-γ in vaccinated people (Fig. 9E) differently to non-vaccinated ones (see Additional file: Fig. 23*,* data availability section).

**Fig. 10:**
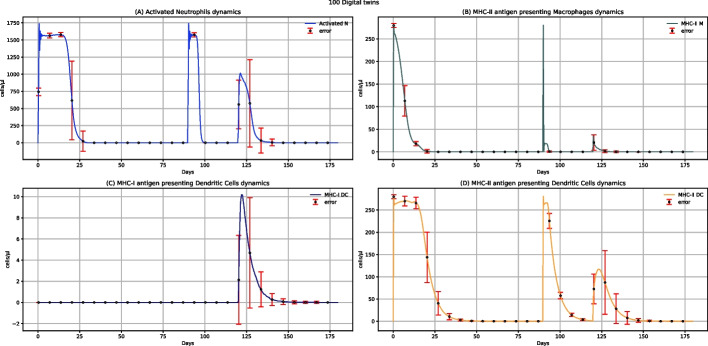
UISS-FLU in silico prediction of activated neutrophils, MHC-II antigen-presenting macrophages, MHC-I antigen-presenting dendritic cells, and MHC-II antigen-presenting dendritic cells population levels. Population averaged immune system dynamics after the first dose of the multi-epitope vaccine administered at time 0, the second at time 40, and the challenge exposure to the H5N1 virus at 120 days are depicted. Red error bars represent the standard deviation

Figure 10 depicts the prediction of activated neutrophils, MHC-II antigen-presenting macrophages, MHC-I antigen-presenting dendritic cells, and MHC-II antigen-presenting dendritic cells dynamic levels after a first dose of the multi-epitope vaccine administrated at time 0, a second dose at time 40, and a challenge exposure with H5N1 virus at 120 days. Comparing the results to the ones depicting the dynamics of the same cells without influenza challenge (Fig. 5), except for MHC-I antigen-presenting dendritic cells, another peak at time 120 days, less high, is present. Similarly, comparing the levels of these cells in vaccinated patients challenged with the H5N1 virus rather than in unvaccinated ones (see Additional file: Fig. 24*,* data availability section), we can see a substantial reduction in the number of cells in vaccinated people. This evidence sheds light on how the vaccine can modulate and reduce the response to the viral infection. In addition, MHC-I antigen-presenting dendritic cells levels are equal to 0 after administering two doses of multi-epitope vaccine (Fig. 5C). At the same time, a peak at around 120 days can be observed in vaccinated patients after challenge exposure to the H5N1 virus (Fig. 10C). This means that the activation of these cells depends on the infection and cannot be attributed to the vaccine administration.

**Fig. 11:**
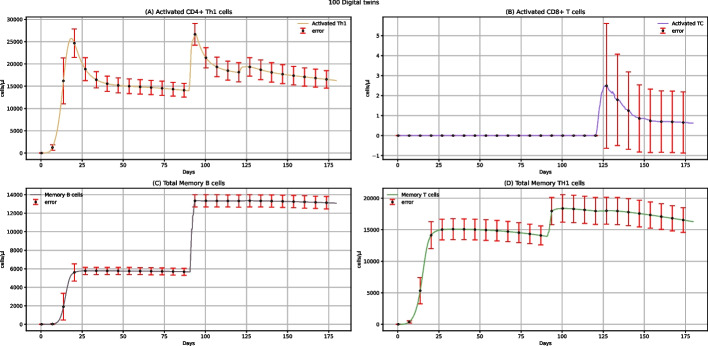
UISS-FLU in silico prediction of activated CD4 + Th1 cells, activated CD8 + T cells, total memory B cells, and total memory Th1 cells population levels. Population averaged immune system dynamics after the first dose of the multi-epitope vaccine administered at time 0, the second at time 40, and the challenge exposure to the H5N1 virus at 120 days. Red error bars represent the standard deviation

Figure 11 shows the in silico results of activated CD4 + Th1 cells, activated CD8 + T cells, total memory B cells, and total memory Th1 cells after administering two doses of the multi-epitope vaccine and the challenge exposure to the H5N1 virus. In the activated CD8 + T cells dynamics, only a peak at 120 days (Fig. 11B) is present; this can be attributed to the virus activation of CD8 + T cells, as the vaccine does not affect these cells. Next, focusing on the total memory B cells (Fig. 11C) and total memory Th1 cells (Fig. 11D), we can evaluate the effect of the proposed multi-epitope vaccine. After two vaccine doses, the memory cells population level remains high and constant on time, compared to the memory cells dynamics in unvaccinated people (see Additional file: Fig. 25*,* data availability section), where the number of cells starts to decrease in a few days. This means that with one dose at 0 and a second one 90 days after, the vaccine's protective effect remains unaltered even after 120 days.

**Fig. 12:**
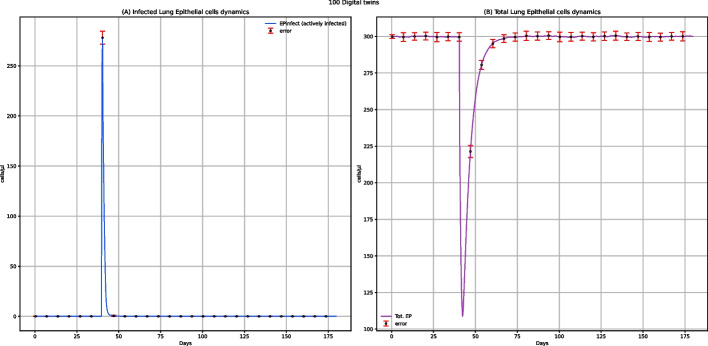
UISS-FLU in silico prediction of infected lung epithelial cells (panel **A**) and total lung epithelial (panel **B**) cells population level. Population averaged immune lung epithelial cells for the in silico unvaccinated cohort after H5N1 influenza virus infection at 40 days is depicted. Red error bars represent the standard deviation

One of the main goals of this work is to evaluate how the designed multi-epitope vaccine can modify the immune response in patients who encounter the H5N1 virus. For this reason, we focused on comparing the results representing the dynamics of lung epithelial cells in vaccinated subjects (Fig. 7) with the unvaccinated ones (Fig. 12) after encountering the H5N1 influenza virus. In Fig. 12B, we can observe that, compared to the initial number of total lung epithelial cells (300 cells/µl), after the H5N1 influenza challenge, there is a reduction of about 180 cells/µl in unvaccinated people. This data appears to agree with experimental data obtained in other studies, according to which the percentage of dead epithelial cells at the peak of the virus titer varied from 37 to 66% [34]. A strong reduction of lung epithelial infected cells in vaccinated patients can be observed. Using UISS-FLU, we simulated several combinations of epitopes, and we focused on the results not only of the best formulation but also of the worst one. We noticed that the number of lung epithelial infected cells in unvaccinated patients (Fig. 12) is similar to the number of the same cells in vaccinated patients with the worst combination we tried (see Additional file: Fig. 46, data availability section) after 120 days from the administration of the vaccine. We can also highlight that the error bar in Fig. 7 is higher in the vaccinated patients than in immune system scenarios without vaccine (Fig. 12); this can reveal interesting insights about the response variability of the effects of the multi-epitope vaccine, while the variability between patients is lower in response to the virus.

#### Assessment of the optimal number of doses to maximize the efficacy of the multi-epitope vaccine

Another aim of this work is to assess how many doses of the multi-epitope vaccine proposed are needed to maintain vaccine protection against H5N1 influenza virus infection and in which timing frame these doses should be administered. Through the UISS-FLU platform, we initially evaluated the effect that the challenge with the H5N1 virus could have after 40, 60, and 120 days from the administration of the best formulation of the multi-epitope vaccine, and we noticed that after 120 days, the protection from the vaccine begins to decrease. For this reason, we tested a booster dose at different time intervals from the first dose. Ultimately, we found that the best formulation has the most prolonged protective effect when administrated in two doses at 0 and 90 days, respectively. In addition, we obtained several results from in silico predictions (see Additional file, in data availability section)*,* and we noticed that the number of lung epithelial infected cells in in silico vaccinated patients with a booster at 90 days is lower than in the ones immunized with a single dose. Furthermore, the number of total epithelial lung cells in virtual patients vaccinated with one dose is lower than in the ones with the booster, confirming that the multi-epitope vaccine better protects when a booster is administered.

Ethical approval from a named institution and written informed consent were not sought as the study does not include any patient or animal.

## Discussion

As expected, a formulation of multi-epitope vaccine with a higher binding affinity score of each epitope should have shown more efficacy; hence we tried a formulation with 0.73, 0.79, and 0.53 affinity binding scores for CTL, Helper-T- lymphocytes, and B-cell epitopes, respectively; on the other hand, we also evaluated another formulation with 0.73, 0.58, and 0.63 affinities binding score. However, conversely to what we expected, based on the result of UISS-FLU, the high binding affinity score is not necessarily correlated to the high efficacy of the vaccine and can be related to the vaccine's antigenicity. For example, the formulation with a higher binding affinity score (0.79) for Helper-T-lymphocytes epitope shows fewer titers of IgG than the formulation with a low binding affinity score for Helper T-lymphocytes epitope. This could be noticed after administering the first dose at time 0 and the influenza virus challenge at day 40.

The evaluation of vaccine efficacy is a complex measurement that illustrates how it works and indicates the type of immune responses the vaccine generates and their magnitude over time. Vaccination aims to develop long-lived immunological protection. The administration of each dose of vaccine leads to an increase in the total number of Th-1 and B-cell memory. A high number of B and T-cell memory in virtual patients who received long-term booster doses is remarkable compared to those just exposed to the H5N1 influenza virus challenge.

Several approaches have been employed to predict vaccine efficacy at the human population level, including mathematical models able to predict the correlation between antibody titers and vaccine efficacy. Among these, a notable study undertook the development of a model capable of simulating the dynamic progression of SARS-CoV-2 infection within the host. Leveraging this model, the researchers conducted simulations utilizing virtual patient populations while incorporating the crucial influence of neutralizing antibody (NAb) responses. By shedding light on these mechanistic foundations, the study offers plausible explanations for the observed efficacy of these vaccines [36]. Another study aimed to find a correlate of protection (CoP) for COVID-19 vaccines. Evaluating antibody titers of seven existing vaccines showed a strong correlation between neutralizing titer and efficacy and binding antibody titer and efficacy. These findings support post-immunization antibody titers for establishing a CoP for COVID-19 vaccines [37].

Moreover, in silico vaccine design frequently does not consider specific steps that lead to the final vaccine formulation. For example, codon optimization and cloning, adjuvant selection, and delivery system selection are usually performed in an in vitro/in vivo settings. However, some examples of toolkits can provide some suggestions within this context. For example, the JCat (Java Codon Adaptation Tool)^7^ server gives insights about a nucleotide sequence, essential properties, including codon adaptation Index (CAI), and information to assess the protein expression in the host. Snapgene represents another example^8^ that can be used to clone the candidate construct to ensure cloning and expression.

Influenza infections start in the upper respiratory part, where lung epithelial cells are infected [38]. For this reason, another important factor in explaining influenza vaccine efficacy is the number of infected lung epithelial cells. The outcome of UISS-FLU demonstrates that the number of infected lung epithelial cells is approximately ten times higher in virtual patients exposed to influenza than in those who received a booster dose.

According to the results obtained from UISS-FLU for all the simulated scenarios and related concentration and population levels of several immunological entities (IL-1, IL-2, IL-6, IL-12, INF-γ, TNF- α, T-cell memory, B-cell memory, number of infected epithelial cells, IgG, IgM, IgA), we suggest that a booster dose of the best formulation of multi-epitope vaccine is required to be administered to obtain long-term protection.

## Conclusion

Reverse vaccinology is the first application of genomic technologies in vaccine design, demonstrating a great revolution in vaccine discovery. It provides a helpful context for researchers to identify protective targets and design an effective vaccine pipeline for pathogens, supporting traditional approaches. Recently, the RV pipeline has been increasingly developed using immunoinformatic tools, which are cost-effective and time-saving in predicting potential antigenic epitopes required for vaccine candidates.

Designed in silico RV pipeline, particularly in an emergency scenario, is beneficial to control future possible endemic or pandemics provoked by climate changes that heavily affect our planet. Indeed, global warming indirectly affects the emergence of new pandemics through melting glaciers and the release of survived viruses. The H5N1 strain of influenza A virus may be one of the sources of a future pandemic because of its presence in frozen lakes. For these reasons, it may be beneficial to have a ready-to-use pipeline for discovering a potential vaccine against the pathogen to accelerate and optimize a new vaccine formulation.

However, the current RV pipeline misses the possibility of evaluating the efficacy of an in silico*-designed* vaccine. To go beyond the RV state of the art, we proposed to use an advanced immune system modeling and simulation platform capable of envisaging the efficacy of the candidate vaccine in a disease-related context of use. As a working example, we showed the concrete application of this extended RV pipeline to the design of a multi-epitope vaccine against H5N1 influenza, also estimating its efficacy. As a result, the in silico*-designed* multi-epitope vaccine consisting of T and B-cell epitopes of HA and NA proteins was found to be antigenic, immunogenic, and effective, based on UISS-FLU outcomes. We can suggest that this vaccine pipeline can be used for different diseases scenario based on their specific pathogens. However, in vivo experimental confirmation and regulatory approval are still needed to confirm the in silico predictions. Unfortunately, experimental confirmation is a time and money-consuming process that is out of our scope, as an *in-silico* team. In this context, using tools and methods based on sequence and structure analysis and prediction, like simulated molecular docking, can help improve prediction accuracy, providing a much clearer understanding of the conformational changes of molecules and peptides to verify the affinity of epitopes.

Furthermore, molecular docking can evaluate the interaction between the HLA allele and selected peptides and T-cell epitopes with the highest binding energy value. Also, dynamic simulation can support the vaccine construct's stability prediction and population coverage analysis. These tools can be used in an integrative environment to provide a good in silico platform for vaccine design. However, all the existing toolkits still lack vaccine efficacy prediction that can now be addressed by employing in silico trial platforms, like the one proposed in this work.

## Data Availability

Additional file can be found following the GitHub public repository link: https://github.com/COMBINE-Group/Beyond-the-state-of-the-art-of-reverse-vaccinology-predicting-vaccine-efficacy-with-UISS-FLU.git.

## Footnotes

1 https://services.healthtech.dtu.dk/service.php?NetCTL-1.2

2 https://services.healthtech.dtu.dk/service.php?NetMHCIIpan-4.0

3 http://tools.iedb.org/bcell/

4 https://www.efsa.europa.eu/en/glossary/allergenicity

5 http://www.ddg-pharmfac.net/vaxijen/VaxiJen/VaxiJen.html

6 https://www.ddg-pharmfac.net/AllerTOP/

7 http://www.prodoric.de/JCat

8 https://www.snapgene.com

## References

[1] Moxon R, Reche PA, Rappuoli R (2019). Editorial: reverse vaccinology. Front Immunol.

[2] Mora M, Veggi D, Santini L, Pizza M, Rappuoli R (2003). Reverse vaccinology. Drug Discov Today.

[3] Castello JD, Rogers SO (2005). Life in ancient ice.

[4] Castello JD, Rogers SO, Starmer W, et al. Detection of tomato mosaic tobamovirus RNA in ancient glacial ice. Polar Biol 1999;22:207–212. 10.1007/s003000050411.

[5] Zhang G, Shoham D, Gilichinsky D, Davydov S, Castello JD, Rogers SO (2006). Evidence of influenza A virus RNA in Siberian Lake ice. J Virol.

[6] Abyzov SS (1993). Microorganisms in the Antarctic ice. Antarct Microbiol.

[7] Simmons GM, Vestal JR, Wharton RA (1993). Environmental regulators of microbial activity in continental Antarctic lakes. Phys Biogeochem Process Antarct Lakes.

[8] Catranis C (1991). Microorganisms entrapped in glacial ice. Antarct J US.

[9] Rogers SO, Starmer WT, Castello JD (2004). Recycling of pathogenic microbes through survival in ice. Med Hypotheses.

[10] Parker Lv, Martel CJ. Long-term survival of enteric microorganisms in frozen wastewater. 2002.

[11] Horimoto T, Kawaoka Y (2001). Pandemic threat posed by avian influenza A viruses. Clin Microbiol Rev.

[12] Webster RG, Bean WJ, Gorman OT, Chambers TM, Kawaoka Y (1992). Evolution and ecology of influenza A viruses. Microbiol Rev.

[13] Le TH, Nguyen NTB (2014). Evolutionary dynamics of highly pathogenic avian influenza A/H5N1 HA clades and vaccine implementation in Vietnam. Clin Exp Vaccine Res.

[14] Sylte MJ, Suarez DL (2009). Influenza neuraminidase as a vaccine antigen. Curr Top Microbiol Immunol.

[15] Russell CJ, Hu M, Okda FA (2018). Influenza hemagglutinin protein stability, activation, and pandemic risk. Trends Microbiol.

[16] Russo G, di Salvatore V, Sgroi G, Parasiliti Palumbo GA, Reche PA, Pappalardo F (2022). A multi-step and multi-scale bioinformatic protocol to investigate potential SARS-CoV-2 vaccine targets. Brief Bioinform.

[17] Pappalardo F, Russo G, Tshinanu FM, Viceconti M. In silico clinical trials: concepts and early adoptions. 10.1093/bib/bby043. 10.1093/bib/bby043 29868882

[18] Palladini A, Nicoletti G, Pappalardo F, Murgo A, Grosso V, Stivani V (2010). In silico modeling and in vivo efficacy of cancer-preventive vaccinations. Cancer Res.

[19] Papparlardo F, Russo G, Pennisi M, Palumbo GAP, Sgroi G, Motta S, et al. The potential of computational modeling to predict disease course and treatment response in patients with relapsing multiple sclerosis. 2020. 10.20944/PREPRINTS202001.0174.V1. 10.3390/cells9030586 PMC7140535 32121606

[20] Pennisi M, Russo G, Sgroi G, Bonaccorso A, Parasiliti Palumbo GA, Fichera E (2019). Predicting the artificial immunity induced by RUTI® vaccine against tuberculosis using universal immune system simulator (UISS). BMC Bioinf.

[21] Pappalardo F, Russo G, Corsini E, Paini A, Worth A (2022). Translatability and transferability of in silico models: context of use switching to predict the effects of environmental chemicals on the immune system. Comput Struct Biotechnol J.

[22] Russo G, Pennisi M, Fichera E, Motta S, Raciti G, Viceconti M (2020). In silico trial to test COVID-19 candidate vaccines: a case study with UISS platform. BMC Bioinf.

[23] Pappalardo F, Forero IM, Pennisi M, Palazon A, Melero I, Motta S (2011). SimB16: modeling induced immune system response against B16-melanoma. PLoS ONE.

[24] Sun B, Zhang J, Li Z, Xie M, Luo C, Wang Y, et al. Integration: gospel for immune bioinformatician on epitope-based therapy. Front Immunol. 2023;14. 10.3389/fimmu.2023.1075419 PMC9927647 36798136

[25] Sievers F, Wilm A, Dineen D, Gibson TJ, Karplus K, Li W (2011). Fast, scalable generation of high-quality protein multiple sequence alignments using Clustal Omega. Mol Syst Biol.

[26] Procter JB, Carstairs GM, Soares B, Mourão K, Ofoegbu TC, Barton D, et al. Alignment of biological sequences with Jalview. In: Methods in molecular biology. Humana Press Inc.; 2021. p 203–24. 10.1007/978-1-0716-1036-7_13 PMC7116599 33289895

[27] Stanekov Z, Varekov E (2010). Conserved epitopes of influenza A virus inducing protective immunity and their prospects for universal vaccine development. Virol J.

[28] Reynisson B, Barra C, Kaabinejadian S, Hildebrand WH, Peters B, Nielsen M (2020). Improved prediction of MHC II antigen presentation through integration and motif deconvolution of mass spectrometry MHC eluted ligand data. J Proteome Res.

[29] Schonlau M, Zou RY (2020). The random forest algorithm for statistical learning. Stata Journal.

[30] Zhang J, Tao A, Tao A, Raz E, Bioinformatics A, bioinformatics T. Antigen Immunogen Allergen. 2015;175–86.

[31] Dimitrov I, Bangov I, Flower DR, Doytchinova I (2014). AllerTOP vol 2–a server for in silico prediction of allergens. J Mol Model.

[32] Juárez MA, Pennisi M, Russo G, Kiagias D, Curreli C, Viceconti M (2020). Generation of digital patients for the simulation of tuberculosis with UISS-TB. BMC Bioinf.

[33] Russo G, Pennisi M, Fichera E, Motta S, Raciti G, Viceconti M (2020). In silico trial to test COVID-19 candidate vaccines: a case study with UISS platform. BMC Bioinf.

[34] Baccam P, Beauchemin C, Macken CA, Hayden FG, Perelson AS (2006). Kinetics of influenza A Virus infection in humans. J Virol.

[35] Luheshi G (1998). Cytokines and fever: mechanisms and sites of action. Ann N Y Acad Sci.

[36] Padmanabhan P, Desikan R, Dixit NM (2022). Modeling how antibody responses may determine the efficacy of COVID-19 vaccines. Nat Comput Sci.

[37] Earle KA, Ambrosino DM, Fiore-Gartland A, Goldblatt D, Gilbert PB, Siber GR (2021). Evidence for antibody as a protective correlate for COVID-19 vaccines. Vaccine.

[38] Klomp M, Ghosh S, Mohammed S, Khan MN. From virus to inflammation, how influenza promotes lung damage. 10.1002/JLB.4RU0820-232R. 10.1002/JLB.4RU0820-232R PMC7937770 32895987

